# Garnet-type Na_3_Te_2_(FeO_4_)_3_

**DOI:** 10.1107/S2056989023002293

**Published:** 2023-03-15

**Authors:** Felix Eder, Matthias Weil

**Affiliations:** a Institute for Chemical Technologies and Analytics, Division of Structural Chemistry, TU Wien, Getreidemarkt 9/E164-05-01, A-1060 Vienna, Austria; University of Kentucky, USA

**Keywords:** crystal structure, garnet, oxidotellurate(VI), isotypism, structural similarity

## Abstract

Na_3_Te_2_(FeO_4_)_3_ or Na_3_Te_2_Fe_3_O_12_, tris­odium ditellurium(VI) triiron(III) dodeca­oxide, was obtained in the form of single-crystals under hydro­thermal conditions. Na_3_Te_2_(FeO_4_)_3_ adopts the garnet structure type in space group *Ia*  *d* and comprises one Na (multiplicity 24, Wyckoff letter *c*, site symmetry 2.22), one Te (16 *a*, .  .), one Fe (24 *d*,  ..) and one O atom (96 *h*, 1) in the asymmetric unit. The three-dimensional framework structure is built of [TeO_6_] octa­hedra and [FeO_4_] tetra­hedra by vertex-sharing. The larger Na^+^ cations are situated in the inter­stices of the framework and are eightfold coordinated in the form of a distorted dodeca­hedron. Qu­anti­tative structural comparisons with isotypic Na_3_Te_2_[(Fe_0.5_Al_0.5_)O_4_]_3_ and Na_3_Te_2_(GaO_4_)_3_ show a high degree of similarity between the three crystal structures.

## Abstract 2

The garnet-type crystal structure of Na_3_Te_2_(FeO_4_)_3_ shows high similarities with its isotypic analogues Na_3_Te_2_[(Fe_0.5_Al_0.5_)O_4_]_3_ and Na_3_Te_2_(GaO_4_)_3_.

## 1. Chemical context

Layered oxidotellurates(VI) comprising an alkali metal (or ammonium) and a transition metal *M*, such as (NH_4_)_4_(VO_2_)_2_Te_2_O_8_(OH)_2_·2H_2_O **(**Nagarathinam *et al.*, 2022 ▸), Li_2_Ni_2_TeO_6_ (Grundish *et al.*, 2019 ▸), Na_3_Ni_1.5_TeO_6_ (Grundish *et al.*, 2020 ▸) or K_2_ *M*_2_TeO_6_ (*M* = Ni, Mg, Zn, Co, Cu; Masese *et al.*, 2018 ▸) are considered to be promising battery materials. In the quest for new representatives of this group of materials comprising K and Fe^III^, we obtained a phase under hydro­thermal conditions with a supposed composition of K_12_Fe^III^_6_Te^VI^_4_O_27_·3H_2_O. However, this phase is not layered but crystallizes in a cubic framework structure with positionally disordered crystal water mol­ecules [*Z* = 4, space group *I* 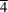 3*d*, *a* = 14.7307 (12) Å at room temperature; Eder & Weil, 2023 ▸], which is closely related to the phase K_12+6*x*_Fe_6_Te_4–*x*_O_27_ [*x* = 0.222 (4), *Z* = 4, space group *I* 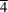 3*d, a* = 14.7440 (10) Å at 100 K; Albrecht *et al.*, 2021 ▸]. With the intention of synthesizing the possible Na-analogue Na_12_Fe^III^_6_Te^VI^_4_O_27_·3H_2_O, we obtained garnet-type Na_3_Te_2_(FeO_4_)_3_ instead, and report here its crystal structure and qu­anti­tative comparisons with related crystal structures.

## 2. Structural commentary

**Figure 1:**
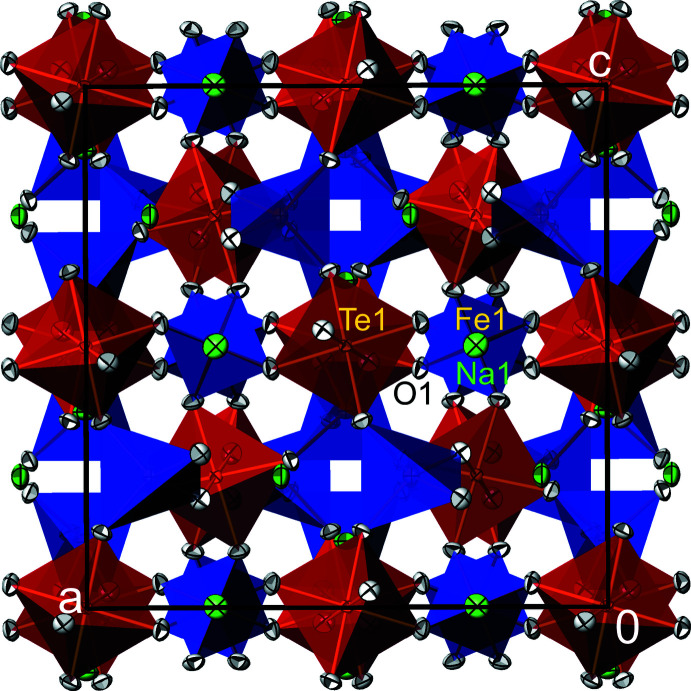
Projection of the garnet-type crystal structure of Na_3_Te_2_(FeO_4_)_3_ along [0 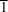 0]. Displacement ellipsoids are drawn at the 90% probability level. [TeO_6_] octa­hedra (red) and (FeO_4_) tetra­hedra (blue) are given in the polyhedral representation, Na atoms as green ellipsoids and O atoms as white ellipsoids.

The garnet supergroup has the general formula {*X*_3_}[*Y*_2_](*Z*_3_)*φ*_12_ and includes all phases, which crystallize isostructurally with garnet, regardless of the type of elements present at the four atomic sites (Grew *et al.*, 2013 ▸). The crystal structure of garnet comprises a three-dimensional framework built of [*Yφ*_6_] octa­hedra and (*Z*φ_4_) tetra­hedra in which each octa­hedron is joined to six others through vertex-sharing tetra­hedra. In turn, each tetra­hedron shares its vertices with four octa­hedra, so that the composition of the framework is *Y*_2_ *Z*_3_ *φ*_12_. Larger *X* atoms occupy positions in the inter­stices of the framework and are eightfold coordinated in the form of a distorted dodeca­hedron (Wells, 1975 ▸). In a crystal–chemical sense, the final composition can therefore be expressed as {*X*_3_}^[8do]^[*Y*_2_]^[6o]^(*Z*_3_^[4t]^)φ_12_, or as {*X*_3_}^[8do]^[*Y*_2_]^[6o]^(*Z* ^[4t]^ *φ*_4_)_3_. In the title compound, Na takes the *X* position (multiplicity 24, Wyckoff letter *c*, site symmetry 2.22), Te the *Y* position (16 *a*, . 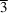 .), Fe the *Z* position (24 *d*, 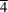 ..) and O the *φ* position (96 *h*, 1). The crystal structure of Na_3_Te_2_(FeO_4_)_3_ is displayed in Fig. 1 ▸. Bond-valence sums (Brown, 2002 ▸) for all atoms were computed with the parameters of Brese & O’Keeffe (1991 ▸). The values (in valence units) of 1.19 for Na, 6.00 for Te, 2.98 for Fe and 2.04 for O are in very good agreement with the expected values of 1, 6, 3 and 2, respectively.

The garnet supergroup includes several chemical classes, which is also reflected by the high number of phases that adopt the garnet structure type. A search in the ICSD (version 2022-1; Zagorac *et al.*, 2019 ▸), using the garnet structure type in space group *Ia* 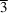 *d* and with Si on the *Z* position as search field revealed about 420 entries, and with atoms other than Si on the *Z* position about 350 entries. With Te on the *Y* position, only five phases were found, including the mineral yafsoanite [ideally Ca_3_Te_2_(ZnO_4_)_3_, Jarosch & Zemann, 1989 ▸; Mills *et al.*, 2010 ▸], the Li-conducting Nd_3_(Te_2–*x*_Sb_*x*_)(Li_3+*x*_O_4_)_3_ (*x* = 0.05, 0.10) (O’Callaghan *et al.*, 2008 ▸), Na_3_Te_2_[(Fe_0.5_Al_0.5_)O_4_]_3_ (Wedel & Sugiyama, 1999 ▸) and Na_3_Te_2_(GaO_4_)_3_ (Frau *et al.*, 2008 ▸). The latter two phases comprise Na on the *X* position and, with respect to the title compound, therefore are the chemically most related compounds. A comparison of relevant bond lengths in the three garnets, together with structural similarity parameters, as revealed by the program *compstru* (de la Flor *et al.*, 2016 ▸) available at the Bilbao Crystallographic Server (Aroyo *et al.*, 2006 ▸), is given in Table 1 ▸. The cations occupying the *Z* site apparently influence the two Na—O bond lengths in the crystal structures, although the ionic radii (Shannon, 1976 ▸) of *Z* do not directly correlate with this behaviour. The title compound with *Z* = Fe (ionic radius 0.49 Å) has the longest Na—O bonds, followed by the mixed-occupied compound with *Z* = (Fe,Al) (averaged ionic radius 0.44 Å) and the compound with *Z* = Ga (ionic radius 0.47 Å). On the other hand, the Te—O bond lengths in the three garnet structures are virtually identical.

An X-ray powder diffraction pattern of Na_3_Te_2_(FeO_4_)_3_ has been deposited with the ICDD (PDF 00-048-0300; Gates-Rector & Blanton, 2019 ▸) without giving atomic coordinates for the O-atom site or displacement parameters for the atoms. The corresponding unit-cell parameter *a* = 12.5257 (1) Å determined from room-temperature powder X-ray measurement data is in very good agreement with the one from single-crystal data (Table 2 ▸). In the context of investigating the magnetic ordering of Fe^III^ on the *Z* sites, neutron powder data recorded at room temperature were also reported for Na_3_Te_2_(FeO_4_)_3_ (Plakhtii *et al.*, 1977 ▸).

## 3. Synthesis and crystallization

The solid educts Fe(NO_3_)_3_·9H_2_O, TeO_2_, H_6_TeO_6_ and NaOH were weighed in the molar ratios 2:1:2:15 and placed into a Teflon container (inner volume *ca* 5 ml). The container was filled to about 2/3 of its volume with water, closed with a Teflon lid and embedded into a steel autoclave. The hydro­thermal experiment was conducted at 473 K for five days. The solid product was filtered off, washed with water and ethanol and dried in air. It consisted of light-brown microcrystalline material and a few amber-coloured cuboid crystals of Na_3_Te_2_(FeO_4_)_3_, as well as a very few small yellowish platy crystals of an unknown phase. Preliminary single-crystal measurements of the latter indicated a unit cell with hexa­gonal metrics (*a* = 5.252, *c* = 15.724 Å) and obvious twinning, which has precluded a structure solution so far. Similar metrics were found for Na_2_GeTeO_6_ (Woodward *et al.*, 1998 ▸). The powder X-ray diffraction pattern of the bulk revealed Na_3_Te_2_(FeO_4_)_3_ as a side product and the unknown phase (assuming a close relation with Na_2_GeTeO_6_) as the main phase, in an approximate mass ratio of 0.15:0.85.

## 4. Refinement

Crystal data, data collection and structure refinement details are summarized in Table 2 ▸.

## Supplementary Material

Crystal structure: contains datablock(s) I, global. DOI: 10.1107/S2056989023002293/pk2684sup1.cif

Structure factors: contains datablock(s) I. DOI: 10.1107/S2056989023002293/pk2684Isup2.hkl

CCDC reference: 2247314

Additional supporting information:  crystallographic information; 3D view; checkCIF report

## Figures and Tables

**Table 1:** Selected bond lengths (Å) in related garnet-type Na_3_Te_2_(*Z*O_4_)_3_ oxidotellurates(VI) and their structure similarity parameters relative to Na_3_Te_2_(FeO_4_)_3_

	Na_3_Te_2_(FeO_4_)_3_	Na_3_Te_2_[(Al,Fe)O_4_]_3_	Na_3_Te_2_(GaO_4_)_3_
Na1—O1 (4×)	2.4208 (10)	2.396 (3)	2.3907 (17)
Na1—O1 (4×)	2.6226 (10)	2.597 (3)	2.5609 (17)
Te1—O1 (6×)	1.9169 (9)	1.914 (2)	1.9124 (17)
*M*1—O1 (4×)	1.8680 (9)	1.829 (2)	1.8405 (16)
			
Degree of lattice distortion, *S*		0.0064	0.0079
Atomic displacement of O1^*a*^ (Å)		0.0205	0.0322
Measure of similarity, Δ		0.001	0.002

Note: (*a*) The three other atomic sites do not show a displacement due to their site symmetries.

**Table 2:** Experimental details

Crystal data	
Chemical formula	Na_3_Te_2_Fe_3_O_12_
*M*_r_	683.72
Crystal system, space group	Cubic, *I* *a* 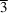 *d*
Temperature (K)	296
*a* (Å)	12.5276 (9)
*V* (Å^3^)	1966.1 (4)
*Z*	8
Radiation type	Mo *K*α
μ (mm^−1^)	10.39
Crystal size (mm)	0.06 × 0.06 × 0.06

Data collection	
Diffractometer	Bruker APEXII CCD
Absorption correction	Multi-scan (*SADABS*; Krause *et al.*, 2015 ▸)
*T*_min_, *T*_max_	0.677, 0.748
No. of measured, independent and observed [*I* > 2σ(*I*)] reflections	42303, 569, 446
*R*_int_	0.060
(sin θ/λ)_max_ (Å^−1^)	0.934

Refinement	
*R*[*F* ^2^ > 2σ(*F* ^2^)], *wR*(*F* ^2^), *S*	0.017, 0.041, 1.16
No. of reflections	569
No. of parameters	18
Δρ_max_, Δρ_min_ (e Å^−3^)	1.25, −0.68

Computer programs: *APEX3* and *SAINT* (Bruker, 2016 ▸), *SHELXT* (Sheldrick, 2015*a* ▸), *SHELXL* (Sheldrick, 2015*b* ▸), *ATOMS for Windows* (Dowty, 2006 ▸) and *publCIF* (Westrip, 2010 ▸).

## supplementary crystallographic information

### Crystal data

**Table:**

Na_3_Te_2_Fe_3_O_12_	Mo *K*α radiation, λ = 0.71073 Å
*M**_r_* = 683.72	Cell parameters from 6128 reflections
Cubic, *I**a*3*d*	θ = 4.0–41.1°
*a* = 12.5276 (9) Å	µ = 10.39 mm^−^^1^
*V* = 1966.1 (4) Å^3^	*T* = 296 K
*Z* = 8	Cube, amber
*F*(000) = 2488	0.06 × 0.06 × 0.06 mm
*D*_x_ = 4.620 Mg m^−^^3^	

### Data collection

**Table:**

Bruker APEXII CCD diffractometer	446 reflections with *I* > 2σ(*I*)
ω– and φ–scans	*R*_int_ = 0.060
Absorption correction: multi-scan (*SADABS*; Krause *et al.*, 2015)	θ_max_ = 41.6°, θ_min_ = 4.0°
*T*_min_ = 0.677, *T*_max_ = 0.748	*h* = −23→23
42303 measured reflections	*k* = −23→23
569 independent reflections	*l* = −23→23

### Refinement

**Table:**

Refinement on *F*^2^	0 restraints
Least-squares matrix: full	*w* = 1/[σ^2^(*F*_o_^2^) + (0.0169*P*)^2^ + 1.9053*P*] where *P* = (*F*_o_^2^ + 2*F*_c_^2^)/3
*R*[*F*^2^ > 2σ(*F*^2^)] = 0.017	(Δ/σ)_max_ < 0.001
*wR*(*F*^2^) = 0.041	Δρ_max_ = 1.25 e Å^−^^3^
*S* = 1.16	Δρ_min_ = −0.68 e Å^−^^3^
569 reflections	Extinction correction: SHELXL-2019/2 (Sheldrick 2015b), Fc^*^=kFc[1+0.001xFc^2^λ^3^/sin(2θ)]^-1/4^
18 parameters	Extinction coefficient: 0.00158 (6)

### Special details

**Table:**

Geometry. All esds (except the esd in the dihedral angle between two l.s. planes) are estimated using the full covariance matrix. The cell esds are taken into account individually in the estimation of esds in distances, angles and torsion angles; correlations between esds in cell parameters are only used when they are defined by crystal symmetry. An approximate (isotropic) treatment of cell esds is used for estimating esds involving l.s. planes.

### Fractional atomic coordinates and isotropic or equivalent isotropic displacement parameters (Å^2^)

**Table:**

	*x*	*y*	*z*	*U*_iso_*/*U*_eq_	
Na1	0.250000	0.375000	0.500000	0.01227 (18)	
Te1	0.500000	0.500000	0.500000	0.00480 (5)	
Fe1	0.250000	0.625000	0.500000	0.00637 (7)	
O1	0.35650 (7)	0.53021 (8)	0.45633 (8)	0.00976 (16)	

### Atomic displacement parameters (Å^2^)

**Table:**

	*U*^11^	*U*^22^	*U*^33^	*U*^12^	*U*^13^	*U*^23^
Na1	0.0150 (3)	0.0068 (4)	0.0150 (3)	0.000	0.0012 (4)	0.000
Te1	0.00480 (5)	0.00480 (5)	0.00480 (5)	0.00038 (3)	0.00038 (3)	0.00038 (3)
Fe1	0.00652 (9)	0.00605 (14)	0.00652 (9)	0.000	0.000	0.000
O1	0.0068 (4)	0.0106 (4)	0.0119 (4)	0.0023 (3)	−0.0013 (3)	−0.0006 (3)

### Geometric parameters (Å, º)

**Table:**

Na1—O1^i^	2.4208 (10)	Te1—O1^viii^	1.9169 (9)
Na1—O1^ii^	2.4208 (10)	Te1—O1^ix^	1.9169 (9)
Na1—O1^iii^	2.4208 (10)	Te1—O1^x^	1.9169 (9)
Na1—O1	2.4208 (10)	Te1—O1^vii^	1.9169 (9)
Na1—O1^iv^	2.6226 (10)	Te1—O1^xi^	1.9169 (9)
Na1—O1^v^	2.6226 (10)	Fe1—O1^xii^	1.8680 (9)
Na1—O1^vi^	2.6226 (10)	Fe1—O1^ii^	1.8680 (9)
Na1—O1^vii^	2.6226 (10)	Fe1—O1^xiii^	1.8680 (9)
Te1—O1	1.9169 (9)	Fe1—O1	1.8680 (9)
			
O1^i^—Na1—O1^ii^	153.42 (4)	O1—Te1—O1^viii^	91.50 (4)
O1^i^—Na1—O1^iii^	73.12 (4)	O1—Te1—O1^ix^	88.50 (4)
O1^ii^—Na1—O1^iii^	113.33 (4)	O1^viii^—Te1—O1^ix^	180.0
O1^i^—Na1—O1	113.33 (4)	O1—Te1—O1^x^	91.50 (4)
O1^ii^—Na1—O1	73.12 (4)	O1^viii^—Te1—O1^x^	88.50 (4)
O1^iii^—Na1—O1	153.42 (4)	O1^ix^—Te1—O1^x^	91.50 (4)
O1^i^—Na1—O1^iv^	125.56 (2)	O1—Te1—O1^vii^	88.50 (4)
O1^ii^—Na1—O1^iv^	77.63 (3)	O1^viii^—Te1—O1^vii^	91.50 (4)
O1^iii^—Na1—O1^iv^	63.92 (4)	O1^ix^—Te1—O1^vii^	88.50 (4)
O1—Na1—O1^iv^	94.14 (3)	O1^x^—Te1—O1^vii^	180.0
O1^i^—Na1—O1^v^	94.14 (3)	O1—Te1—O1^xi^	180.0
O1^ii^—Na1—O1^v^	63.92 (4)	O1^viii^—Te1—O1^xi^	88.50 (4)
O1^iii^—Na1—O1^v^	77.63 (3)	O1^ix^—Te1—O1^xi^	91.50 (4)
O1—Na1—O1^v^	125.56 (2)	O1^x^—Te1—O1^xi^	88.50 (4)
O1^iv^—Na1—O1^v^	106.99 (4)	O1^vii^—Te1—O1^xi^	91.50 (4)
O1^i^—Na1—O1^vi^	63.92 (4)	O1^xii^—Fe1—O1^ii^	113.83 (3)
O1^ii^—Na1—O1^vi^	94.14 (3)	O1^xii^—Fe1—O1^xiii^	101.06 (6)
O1^iii^—Na1—O1^vi^	125.56 (2)	O1^ii^—Fe1—O1^xiii^	113.83 (3)
O1—Na1—O1^vi^	77.63 (3)	O1^xii^—Fe1—O1	113.83 (3)
O1^iv^—Na1—O1^vi^	169.86 (4)	O1^ii^—Fe1—O1	101.06 (6)
O1^v^—Na1—O1^vi^	73.94 (4)	O1^xiii^—Fe1—O1	113.83 (3)
O1^i^—Na1—O1^vii^	77.63 (3)	Fe1—O1—Te1	135.38 (5)
O1^ii^—Na1—O1^vii^	125.56 (2)	Fe1—O1—Na1	92.91 (4)
O1^iii^—Na1—O1^vii^	94.14 (3)	Te1—O1—Na1	107.08 (4)
O1—Na1—O1^vii^	63.92 (4)	Fe1—O1—Na1^ix^	116.33 (4)
O1^iv^—Na1—O1^vii^	73.94 (4)	Te1—O1—Na1^ix^	99.78 (4)
O1^v^—Na1—O1^vii^	169.86 (4)	Na1—O1—Na1^ix^	98.95 (3)
O1^vi^—Na1—O1^vii^	106.99 (4)		

Symmetry codes: (i) −*z*+3/4, −*y*+3/4, −*x*+3/4; (ii) −*x*+1/2, *y*, −*z*+1; (iii) *z*−1/4, −*y*+3/4, *x*+1/4; (iv) *y*−1/4, −*x*+3/4, *z*+1/4; (v) −*z*+1/2, *x*, −*y*+1; (vi) −*y*+3/4, −*x*+3/4, −*z*+3/4; (vii) *z*, *x*, *y*; (viii) −*y*+1, −*z*+1, −*x*+1; (ix) *y*, *z*, *x*; (x) −*z*+1, −*x*+1, −*y*+1; (xi) −*x*+1, −*y*+1, −*z*+1; (xii) −*z*+3/4, −*y*+5/4, *x*+1/4; (xiii) *z*−1/4, −*y*+5/4, −*x*+3/4.
